# A Rare Case of Umbilical Venous Catheter Malposition

**DOI:** 10.7759/cureus.62740

**Published:** 2024-06-20

**Authors:** Mohammad A Simreen, Faten Awaisheh, Rawand Qtaishat

**Affiliations:** 1 Pediatrics, Queen Rania Hospital for Children, Amman, JOR; 2 Neonatology, Queen Rania Hospital for Children, Amman, JOR; 3 Pediatrics, Queen Rania Hospital, Amman, JOR; 4 Pediatrics, Jordanian Royal Medical Services, Amman, JOR

**Keywords:** neonatal respiratory distress, pleural, indwelling catheter, central line insertion, preterm neonate

## Abstract

Umbilical venous catheterization is a common procedure in the neonatal care unit. Although it is an easy procedure, insertion under suboptimal techniques may have devastating effects on the newborn. We present a rare case of umbilical venous catheter (UVC) malposition complicated with respiratory distress, pleural effusion, and acute kidney injury.

## Introduction

Umbilical venous catheterization is a common procedure in neonatal care. Umbilical venous catheters (UVC) are used for the intravenous administration of medications, fluids, parenteral nutrition, and neonatal resuscitation medications when needed [1]. Any worsening in the respiratory or cardiovascular condition after umbilical venous catheterization should raise the possibility of umbilical venous catheter malposition [2,3]. Most of the neonatal care units use chest radiography to ensure the correct position of the umbilical venous catheter. In this report, we described a premature newborn with an umbilical venous catheter malposition. The case was complicated by pleural effusion and acute kidney injury, but the patient recovered completely after umbilical venous catheter removal.

## Case presentation

**Figure 1:**
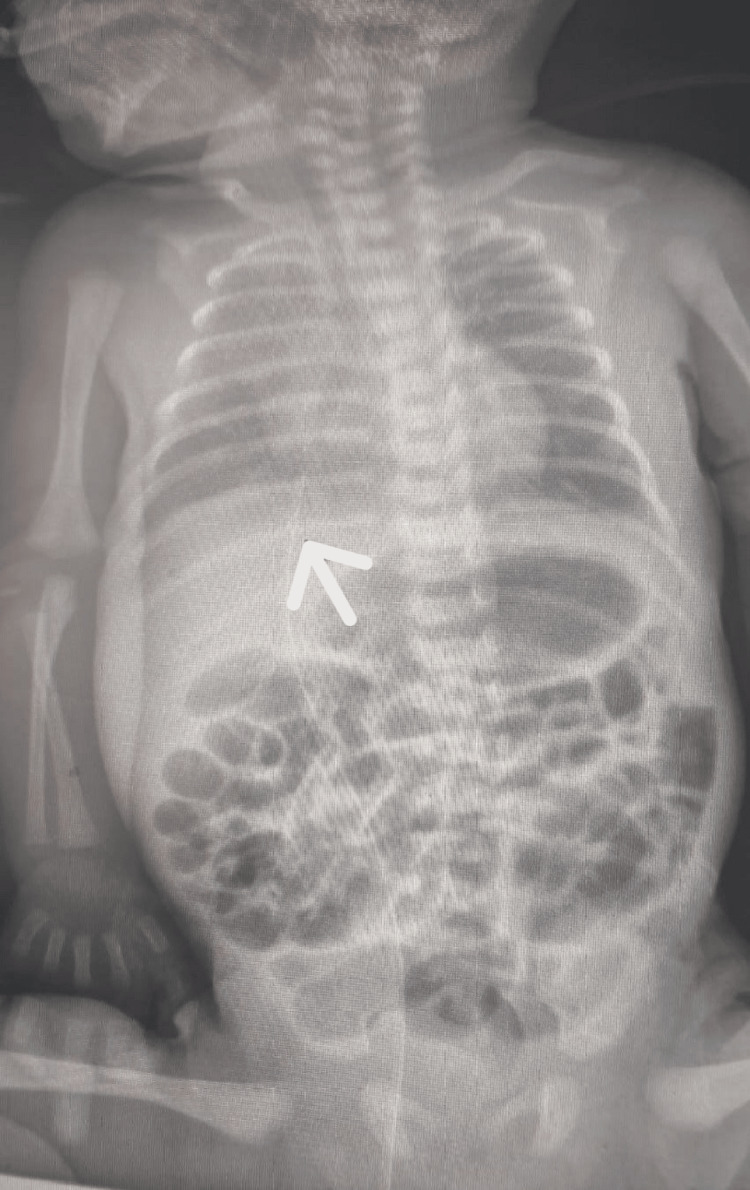
X-ray showing the unrecognized malposition of the umbilical venous catheter

A premature male baby, one of the twins, was born, with a gestational age of 31 weeks and six days and a birth weight of 2.2 kg. He was born to a healthy 34-year-old mother, with an uneventful pregnancy, and delivered via C-section due to failure to progress with a normal appearance, pulse, grimace, activity, and respiration (APGAR) score. He was admitted after birth to the neonatal intensive care unit as a case of respiratory distress syndrome and received two doses of surfactant via endotracheal tube and was maintained on a high-flow nasal cannula. He was referred to our hospital on the second day of life, where an umbilical venous catheter was inserted to continue his intravenous fluids and antibiotics, with respiratory support via high-flow nasal cannula. X-ray was done, which revealed an unrecognized malposition of the umbilical venous catheter (Figure 1).

A complete blood count and kidney function test were within normal ranges initially (Table 1).

**Table 1:** Blood test readings of the neonate during his admission period *Source: [4] ALT, alanine transaminase; AST, aspartate transaminase; BUN, blood urea nitrogen

Blood test	Day 2 (admission)	Day 3	Day 4	Day 5	Day 6	Day 7	Day 10	Reference ranges*	Unit
White blood count	7.6	13.3	11.9	11.6	9	10.6	13.3	5-21	10^3^/μL
Hematocrit	36.5	40	34.9	29.6	29.3	31.9	31.2	42-65	%
Platelets	281	318	129	181	135	169	286	150-450	10^3^/μL
Creatinine	0.67	1.59	1.85	1.34	1.1	0.8	0.42	0.3-1	mg/dL
BUN	12	60	78	66	56	47	9	2-19	mg/dL
Sodium	145	138	136	137	140	139	144	130-145	mEq/L
Potassium	5.9	6.69	7.8	5.2	3.3	5.3	4.7	3.7-5.9	mEq/L
Calcium	9.2	10.8	10.2	9.1	10.5	10.4	10.2	7.5-10	mg/dL
Total bilirubin	7.9	7.9	7.5	10.8	12	11	3.7	<12	mg/dL
Glucose	52	127	119	128	157	129	76	50-90	mg/dL
ALT	8	7	13	25	22	35	20	13-45	U/L
AST	32	28	43	50	45	50	15	47-150	U/L

**Figure 2:**
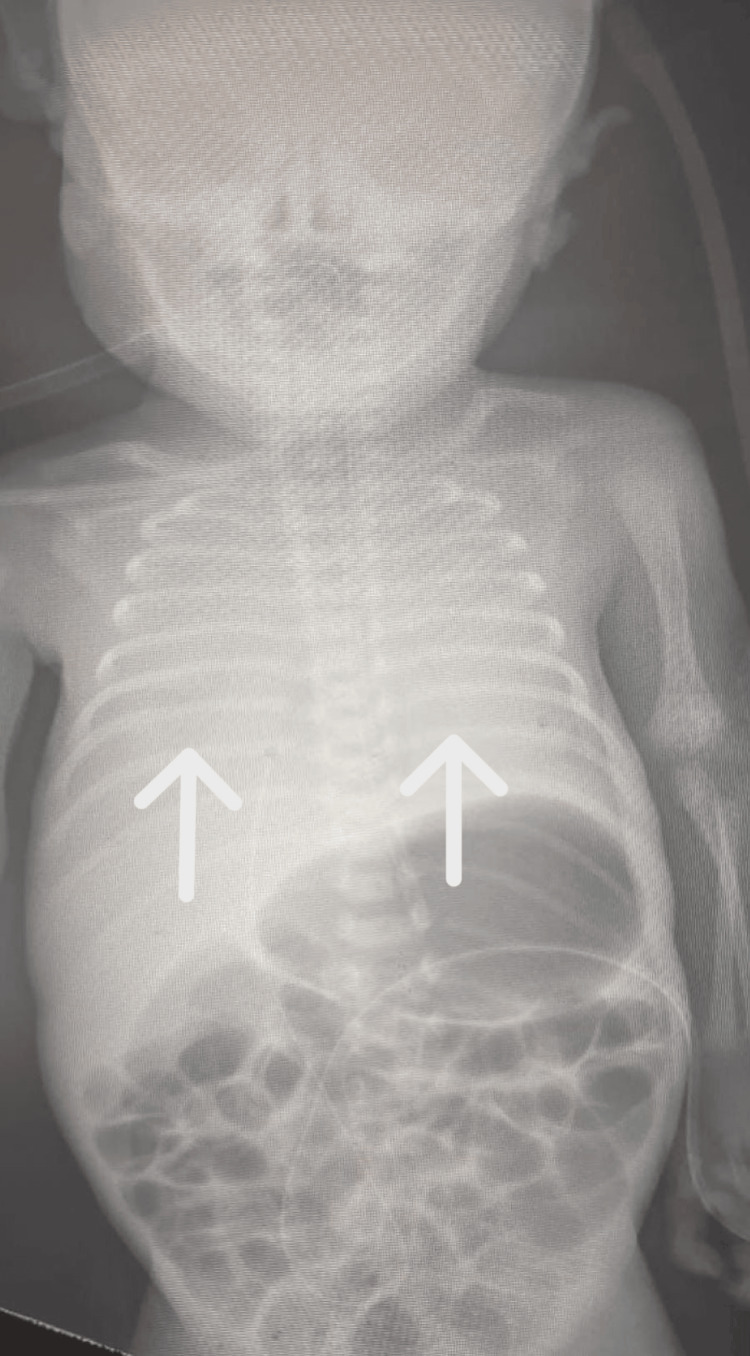
X-ray showing bilateral white lung with an endotracheal tube in the site

On the third day of life, the baby developed desaturation and severe respiratory distress signs when we intubated him and applied him to a mechanical ventilator. Chest X-ray showed a bilateral white lung (Figure 2).

**Figure 3:**
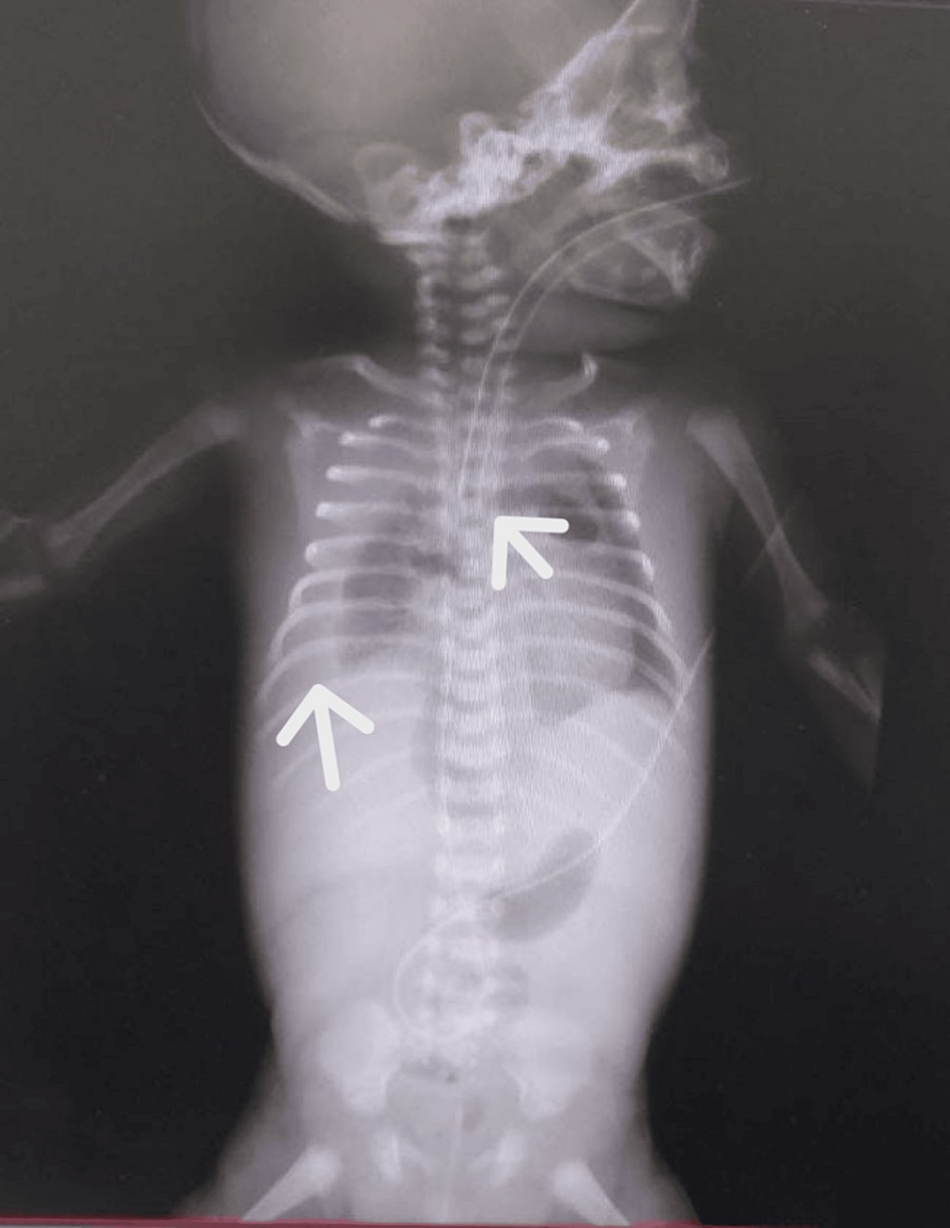
X-ray showing a right-side pleural effusion with an endotracheal tube in the site

Next, he was given a surfactant through the endotracheal tube. On the fourth day, he became dehydrated, and his condition significantly worsened. Chest X-ray revealed a right-side pleural effusion (Figure 3).

**Figure 4:**
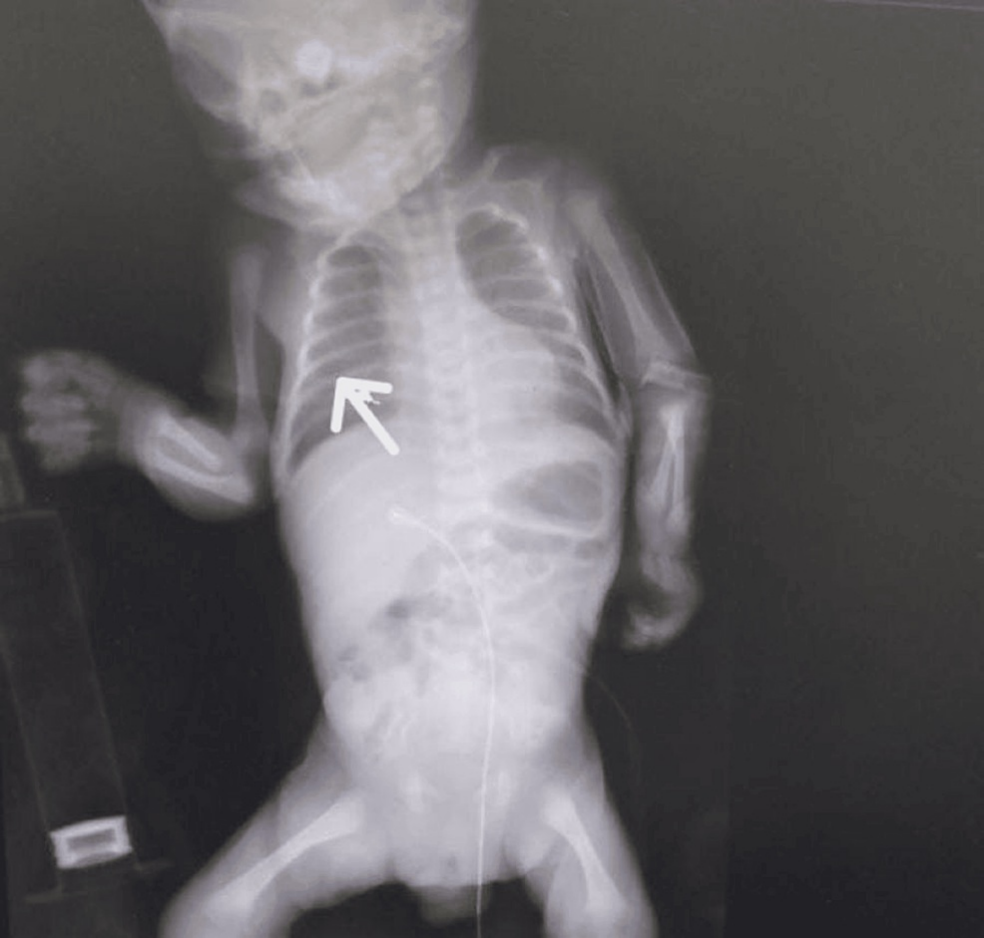
X-ray showing normal lung fields after the removal of the umbilical venous catheter

His blood tests showed high creatinine and urea levels. The umbilical venous catheter was removed, and a peripheral line was inserted to continue his treatment plan. A significant improvement was noticed in his urine output, creatinine level, and general condition. The pleural effusion was resolved without any intervention. After two days, the baby was extubated, and his chest X-ray went back to normal (Figure 4).

Complete improvement in his clinical and laboratory status was seen within the following few days. Then, the baby was discharged home in good health status without O_2_ support.

## Discussion

Umbilical venous catheterization is considered a relatively easy way to obtain central venous access in neonates, especially in premature ones. In our case, with a review of medical literature, it is one of the rarest complications that could happen while inserting the umbilical venous catheter for a neonate. The ideal tip position is at the junction of the inferior vena cava and the right atrium [1]. In most neonatal care centers, position is checked via chest radiography. Based on the radiographic correlation, the optimum tip position is considered to be opposite T8-9 on an anteroposterior film [1].

In a single-center review of cases, pleural effusions were rare at 0.4 per 1000-line days [5]. The most common complication of umbilical venous catheter insertion is malposition [6]. The chances of a misplacement of the catheter are reduced if the catheter is placed under ultrasound guidance [7]. The factors affecting catheter malposition and its complications depend on the insertion technique, size and composition of the catheter, number of manipulations, and contents of parenteral infusion [7]. The migration of an umbilical venous catheter with extravasation to parenteral fluids can also occur and may lead to ascites pleural effusion and pericardial effusion [8].

Determining the correct tip position was found to be more accurate using ultrasound imaging compared to chest radiography, with higher sensitivity and specificity when using ultrasonography as shown in studies [9,10]. Repeated screening by ultrasonography or radiography to assess the correct umbilical venous catheter tip position at regular intervals until removal will help in the early detection of complications [11] or even the avoidance of some of these complications. Although pleural effusion is rare, it can lead to devastating, life-threatening complications. Any worsening in the respiratory or cardiovascular condition after umbilical venous catheterization should raise the possibility of umbilical venous malposition [12,13]. Time management is crucial for better prognosis; hence, pleural effusion should be suspected in any patient with worsening respiratory distress after umbilical venous catheter insertion [12,13].

As mentioned before, the most common complication is malposition [14]. Hepatic extravasation, although uncommon, causes various liver complications (liver abscess, necrosis, and intraparenchymal liver lesions) [15,16]. Other known complications of umbilical venous catheter insertion are catheter-related bloodstream infections, blood loss, thromboembolism, arrhythmias, air embolism, and remnants of a catheter in the umbilicus [15]. Umbilical venous catheters are used for the intravenous administration of medications, fluids, parenteral nutrition, and neonatal resuscitation medications when needed [16]. When malposition is suspected, the immediate removal of the umbilical venous catheter should be done.

## Conclusions

Umbilical venous catheters are important in the management of neonates who are critically ill or premature. Although this procedure is considered relatively easy, the correct tip position should be checked quickly and accurately after insertion. Chest radiography as soon as possible after the insertion of the umbilical venous catheter is considered an adequate way to confirm the position of the catheter, but ultrasonography guidance is more accurate. Serial imaging should be considered after the initial confirmation of the tip position to overcome the risk of the migration of the umbilical venous catheter’s tip. The malposition and migration of the catheter should be considered in any neonate with respiratory and cardiovascular compromise after umbilical venous catheter insertion.
